# Whole-genome sequencing and analysis of *Plasmodium falciparum* isolates from China-Myanmar border area

**DOI:** 10.1186/s40249-018-0493-5

**Published:** 2018-11-13

**Authors:** Hai-Mo Shen, Shen-Bo Chen, Yan-Bing Cui, Bin Xu, Kokouvi Kassegne, Eniola Michael Abe, Yue Wang, Jun-Hu Chen

**Affiliations:** 1 National Institute of Parasitic Diseases, Chinese Center for Disease Control and Prevention; Chinese Center for Tropical Diseases Research; WHO Collaborating Centre for Tropical Diseases, Shanghai, 200025 China; 2 National Center for International Research on Tropical Diseases, Ministry of Science and Technology; Key Laboratory of Parasite and Vector Biology, Ministry of Health, Shanghai, 200025 China; 3 Institute of Parasitic Diseases, Zhejiang Academy of Medical Sciences, Hangzhou, 310013 China; 4 Department of Microbiology and Microbial Engineering, School of Life Science, Fudan University, Shanghai, 200433 China

**Keywords:** *Plasmodium falciparum*, Genome, Variant surface antigen, China-Myanmar border

## Abstract

**Background:**

China has made progress in malaria control and aims to eliminate malaria nationwide, but implementing effective interventions along the border regions remain a huge task. The *Plasmodium falciparum* cases imported from Southeast Asia has frequently reported especially in the China-Myanmar border (CMB) area. Though, information is scant on *P. falciparum* genetic variability in this area.

**Methods:**

This study reported *P. falciparum* isolates genome sequence of six clinical isolates in the CMB area. Furthermore, we estimated the nucleotide diversity, Watterson’s estimator and Tajima’s D value for the whole genome mutation rate in slide window.

**Results:**

Our data were aligned onto 96.05–98.61% of the reference 3D7 genome in high fold coverages. Principal component analysis result showed that *P. falciparum* clustered generally according to their geographic origin. A total of 91 genes were identified as positive selection with Ka/Ks ratio significantly higher than 1, and most of them were multigene families encoding variant surface antigens (VSAs) such as *var*, *rif* and *stevor*. The enrichment of the positive selection on VSA genes implied that the environment complexity subjected CMB’s *P. falciparum* to more pressure for survival.

**Conclusions:**

Our research suggests that greater genetic diversity in CMB area and the positive selection signals in VSA genes, which allow *P. falciparum* to fit the host immune system well and aggravate the difficulty of treatment. Meanwhile, results obtained from this study will provide the fundamental basis for *P. falciparum* population genomic research in CMB area.

**Electronic supplementary material:**

The online version of this article (10.1186/s40249-018-0493-5) contains supplementary material, which is available to authorized users.

## Multilingual abstracts

Please see Additional file 1 for translations of the abstract into the five official working languages of the United Nations.

## Background

Malaria was one of the most prevalent parasitic diseases in the Greater Mekong sub-region (GMS) historically. The GMS countries are Cambodia, China, Laos, Myanmar, Thailand and Vietnam, bounded together by the Mekong River. The area considered covers 2.4 million km^2^ and has a population of about 278 million. Nearly 70% of the local population were at risk of malaria infection [1]. However, decades of control efforts have reduced malaria burden in China from 2961/100 000 population in 1970 to zero indigenous case in 2017 [2, 3], while the goal of nation-wide malaria elimination is rigorously pursued [4]. The situation is different on the other side of the border, because Myanmar is ranked among countries with the highest burden of malaria globally. Previous study conducted passive surveillance for malaria at health facilities along the China-Myanmar border (CMB) area in Yunnan Province to identify risk factors for clinical malaria [5]. It showed that Myanmar does not only share border but also export malaria to China, which seriously impedes the progress of malaria elimination [6]. In addition to the remarkable increase in the number of cases, imported *Plasmodium falciparum* malaria also fuel concerns of re-introduction, and this had been shown to be partly attributed to increases in the intrinsic potential for malaria transmission [7]. Also, antimalarial drug resistance (parasites resistance to chloroquine and pyrimethamine) was first experienced in Southeast Asia before it spread out to Africa. A recent study used in vitro drug assay data from CMB area as phenotypes in genome-wide association study and found several loci associated with in vitro drug resistance to multiple antimalarials [8]. Therefore, there is urgent need to curtail the continued resistance by malaria parasite in the region.

Whole genome studies provide us with the opportunity to develop new control methods, including new drugs and vaccines, improved diagnostics and effective vector control techniques [9]. Several hundreds of *P. falciparum* isolates whole genome had been sequenced in the past, most of them were meant to identify the artemisinin resistance loci [10, 11]. There is paucity of information on whole-genome sequencing of malaria isolates from CMB area. Hence, we performed the whole genome sequencing of *P. falciparum* isolates from CMB to provide information and biological insights that would accelerate the pursuit of effective malaria control in CMB area.

## Methods

### Sampling *P. falciparum* parasites and genome sequencing

High importation risk from Myanmar and wide distribution of malaria vectors in the CMB region sustain risk for secondary infections among local populations. Tengchong County experienced a decreasing malaria prevalence period, and less than 100 confirmed malaria cases were recorded per year from 2011 [12]. Case management of imported malaria within the context of malaria pre-elimination is increasingly considered to be relevant because of the risk of resurgence. Our study assessed the genome sequences of six clinical isolates of *P. falciparum* in the CMB area. Genomic DNA was extracted from the whole blood of malaria cases that were microscopically positive for *P. falciparum* and also confirmed by PCR for single infection. Samples with high parasite density (40 000–260 000 parasites/μl) were selected to ensure the integrity of sequencing. Among hundreds of samples that were collected from CMB area since 2011, only 6 sequenced results were good enough for this study and also provided enough coverage. Genomic DNA was extracted using the QIAGEN DNeasy Blood & Tissue Kit (QIAGEN, Hilden, Germany), and sheared into 350 bp fragments using Covaris instrument. The fragmented DNA molecules were used to construct the sequencing libraries with Illumina TruSeq DNA LT Sample Prep Kit (Illumina, San Diego, USA), and the direct sequencing approach was used in our previous study [13]. We filtered all reads by removing the adapter sequences and low quality sequences with Trimmomatic-3.0 [14]. In addition, the genome and annotation data of the 3D7 reference from PlasmoDB database (http://plasmodb.org) was downloaded [15].

### Identification of SNPs from *P. falciparum* isolates and data analysis

Sequencing reads were mapped from all the six samples of *P. falciparum* 3D7 genome using Burrows-Wheeler Aligner and Sequence Alignment/Map (SAMtools-1.3) [16], which is the most complete whole genome standard reference. The genotyping was performed using an in-house pipeline based on GATK and SnpEff workflows [17]. We performed the principal component analysis (PCA) of all samples and compared the single nucleotide polymorphism (SNP) with those of 34 *P. falciparum* isolates collected worldwide [18, 19]. Then, the nucleotide diversity ($\widehat{\uppi}$), Watterson’s estimator ($\widehat{\theta}$_ω_) for the whole genome mutation rate in 4 kb sliding window and 2 kb step across each chromosome were estimated in ARLEQUIN-Ver3.5 [20]. Also, the Tajima’s D value for each sliding window and the corresponding gene were calculated. Here, the Tajima’s D test help us to distinguish between genes evolving neutrally and under pressure, including selection, demographic expansion or genetic hitchhiking. Genome alignment data were further used to calculate the gene non-synonymous (Ka) and synonymous (Ks) substitution rates with NG (Nei & Gojobori) and YN (Yang and Nielsen) model [21, 22] in KaKs_Calculator1.2 [23], which helps to estimate the balance between neutral mutations, purifying selection and beneficial mutations acting on homologous.

## Results

### Whole genome sequencing of parasites and mapping

**Fig. 1:**
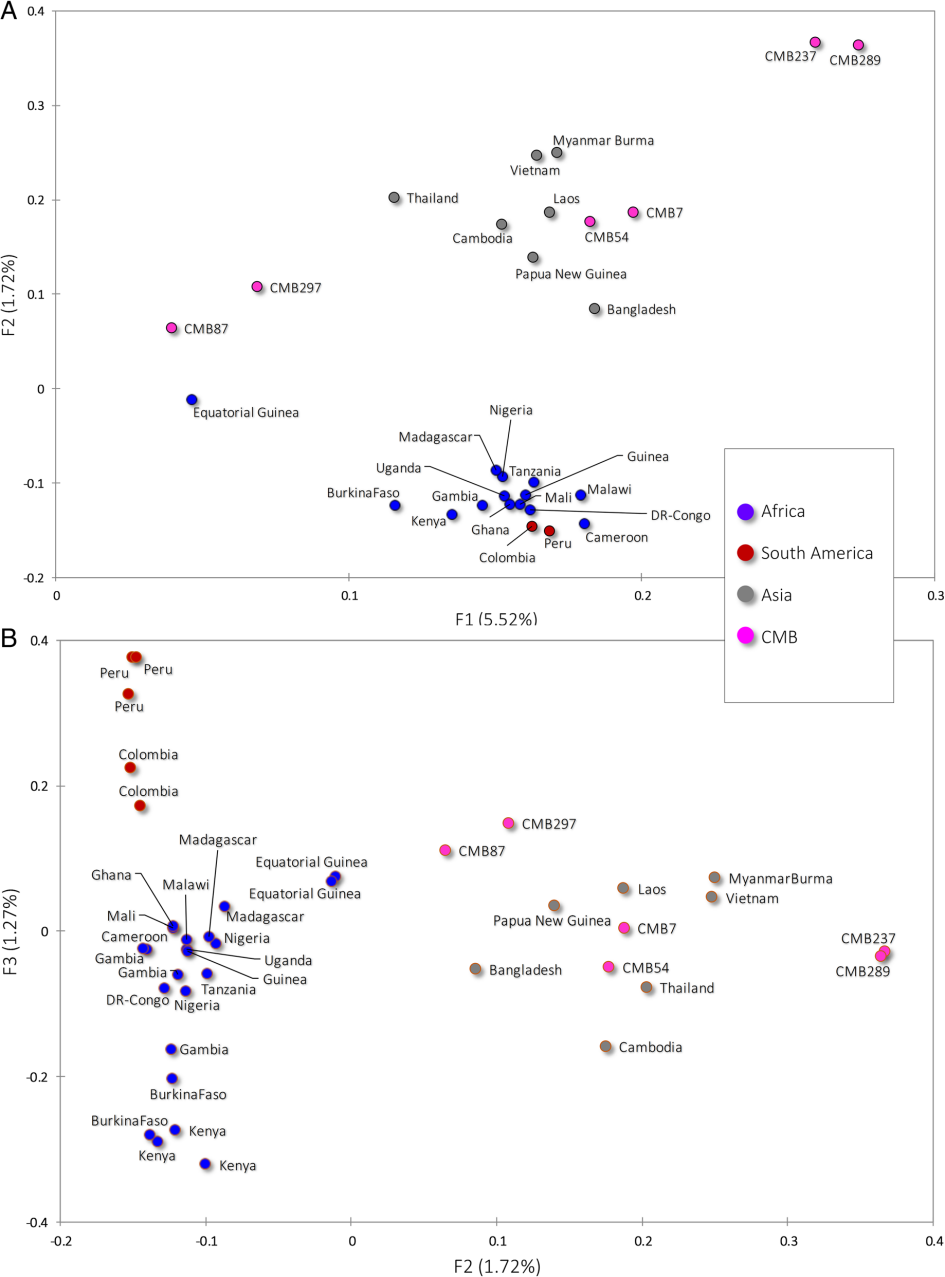
Principal component analysis based on 205 189 common SNP loci in CMB isolates and reference strains. The samples are dyed by their geographic origin: red for South-American, blue for African, grey for Asian, and pink for CMB area. **a**. The major fact (F1) of differentiation showed that *P. falciparum* clustered generally according to their geographic origin. **b**. The second and third facts (F2 and F3) define a distinct South-American cluster and distinguished the African samples better according to their locations

We sequenced the library on Illumina HiSeq X10 (Illumina, San Diego, USA) and generated 78 to 248 M paired-end reads of 150 bp. Illumina sequencing reads have been submitted to the NCBI Short Read Archive (Bio-Project no. PRJNA393218). A variable proportion of reads (4.08–27.08%) from all the isolates were mapped to the reference, and aligned onto 96% of the reference genome in high fold coverage (20.57–121.78×). A total of 369 700 SNPs were captured and only 18 953 common loci were available for analysis after quality filtering (Table 1). PCA of all strains was performed to assess the regional differences. As part of the Asia isolates, the CMB isolates illustrated a higher discrepancies with the 3D7 genome (Fig. 1a). The major axis of differentiation (F1) showed that *P. falciparum* clustered generally according to their geographic origin and the Asian samples exhibited greater genetic diversity than African. The second and third principal components (F2 and F3) defined a distinct South-American cluster and distinguished the African samples better (Fig. 1b). It is important to note that CMB samples were widely separated in our PCA result, suggesting higher diversity from border area.

**Table 1:** Sequencing and mapping summary of 6 isolates of *Plasmodium falciparum* from China-Myanmar border area

Samples	Pf7	Pf54	Pf87	Pf237	Pf289	Pf297
Sequencing and mapping						
Number of clean reads	101 315 479	140 050 984	248 893 994	78 515 613	80 809 032	94 593 018
Mapped on *P. falciparum*	5 430 602	6 342 044	10 150 675	21 265 252	10 626 161	5 929 181
Mapped (%)	5.36	4.53	4.08	27.08	13.15	6.27
Mean mapping quality	47.37	46.59	46.07	53.2	51.98	48.88
Coverage						
Coverage fold	20.57	22.76	35.50	121.78	54.68	23.98
Genome covered > 1 (%)	96.43	96.05	96.96	98.61	98.04	96.10
Variation						
Raw SNP	53 314	50 032	58 753	83 456	70 415	53 730
Filtered SNP	11 838	10 928	606	15 410	14 361	1230
Filtered Indel	15 657	11 474	3094	32 402	27 814	8953

*SNP* single nucleotide polymorphism, *Indel* insertion-deletion

### Scan of important gene under selection

**Fig. 2:**
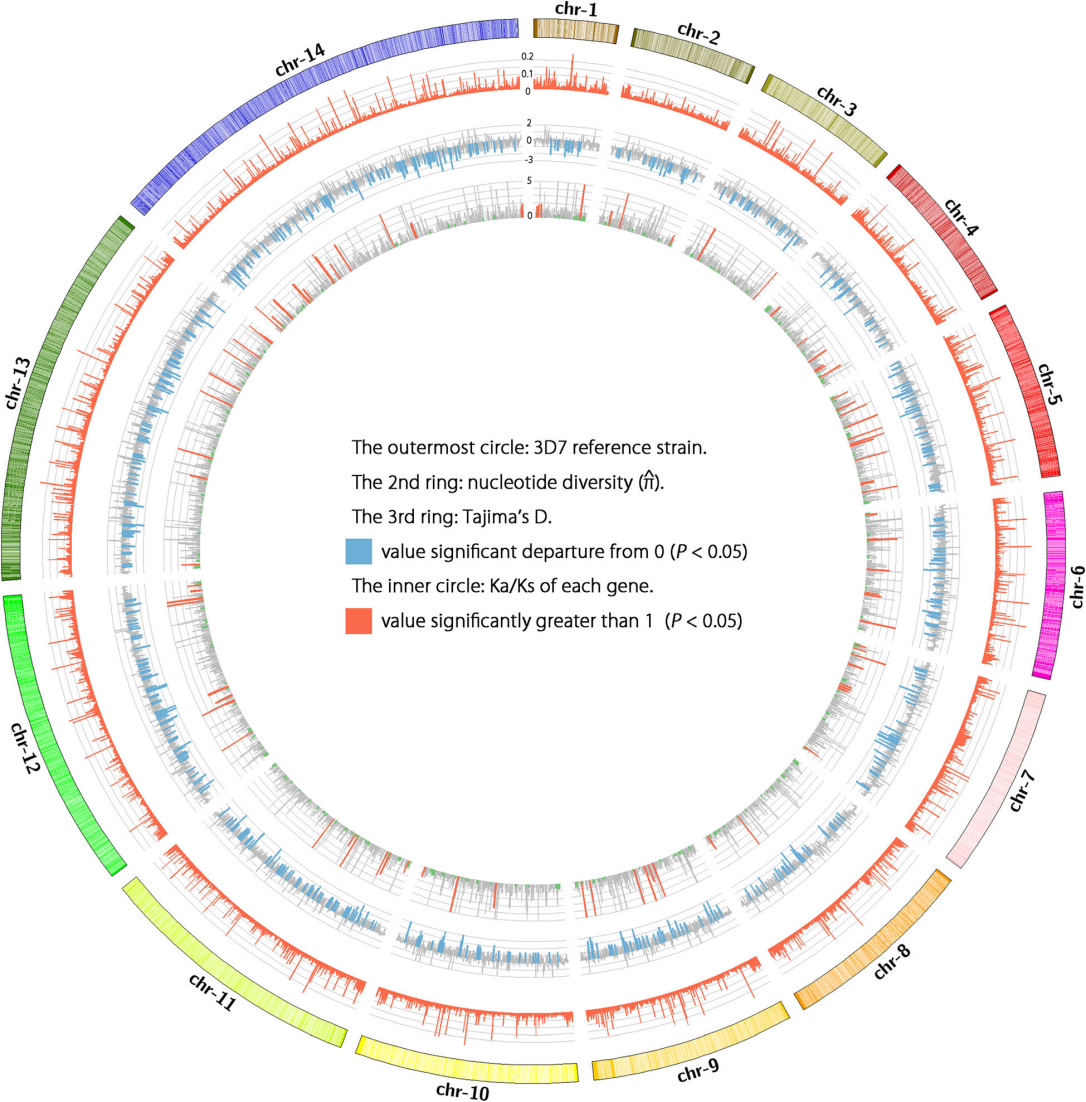
Genomic map of *P. falciparum* CMB isolates. Segments from outside to inside: The outer circle of the diagram depicts the whole genome of 3D7 reference strain and arranged in chromosome order. The second track shows the histogram of $\widehat{\uppi}$ in 4 kb windows. The third track shows the Tajima’s D value. The inner circle shows the Ka/Ks ratio of each gene. This figure was performed using Circos [41]

On genome scale, we estimated $\widehat{\uppi}$ to be 0.0205 and $\widehat{\theta}$_ω_ to be 0.0287, and genetic diversity was lower in exonic regions but higher in intronic and intergenic regions. As expected, the Watterson’s estimator ($\widehat{\theta}$_ω_) is higher than global samples (where $\widehat{\theta}$_ω_ has been estimated to be 1.03 × 10^− 3^ using isolates from Africa, America, Asia and Oceania) [24]. Mean pairwise divergence is higher in gene families associated with red blood cell invasion and immune evasion. The Tajima’s D values obtained were mostly negative, with a mean value of − 1.76, and 103 genes (2.5%) have positive values (Fig. 2).

Of the 5566 genes analyzed, 3485 have valid Ka/Ks value, while only 91 genes were identified as positive selection with Ka/Ks ratio significantly higher than 1 (Fig. 2). However, a high proportion of genes encoding variant surface antigens like the *var* genes, the repetitive interspersed family (*rif*) and the subtelomeric variant open reading frame (*stevor*) was found among these 91 positive selection genes. The variant antigens *P. falciparum* erythrocyte membrane protein 1 (PfEMP1) and repetitive interspersed families of polypeptides (RIFINs) are adhesins implicated in severe *P. falciparum* malaria, and STEVORs are erythrocyte binding protein that mediates merozoite invasion and resetting [25–28].

## Discussion

Recent studies in Myanmar revealed that malaria incidence and mortalities showed significant decreasing trend even in artemisinin-resistant areas [29]. The effective collaboration between China and Myanmar promptly carried out the interventions through simplified processes, and dramatically decreased malaria burden in CMB area [30]. Meanwhile, the relative role of the hidden reservoir of resistant parasites needs to be assessed, particularly in regions that are low-transmission settings and pre-elimination phases [31]. The control of malaria requires intensive efforts, which should be guided by a thorough understanding of adaptive processes occurring in pathogen populations in different endemic areas. Malaria transmission intensity and parasite genetic diversity are known to vary greatly in different parts of Southeast Asia due to variation in rainfall abundance and seasonality [32]. Our result showed that the genetic diversity estimated from CMB *P. falciparum* isolates is higher than the global samples. The genetic structure of CMB parasites was similar with other Asian countries despite the vector species abundance generally enhance the environmental compatibility of parasites [32–35]. Meanwhile, we performed the Tajima’s test to identify genes not fit for the neutral model at equilibrium. These predominantly negative values were remarkably similar to previous analyses, which indicated population expansion of *P. falciparum* in Africa [36]. The greater genetic diversity and selection signals in genes associated with red blood cell invasion and drug resistance are consistent with previous research [37] and also suggested the malaria control programs of Myanmar imposed huge pressure on *P. falciparum* in CMB area and play an important role in the process of diversification.

On the other, it is advantageous to apply positive selection test where environment factors apply consistent pressure over generations in favor of specific beneficial trait. Early analyses identified loci showing evidence of recent positive directional selection and balancing selection confirmed that antimalarial drugs and host immunity have been major selective agents [38]. In our study, genes encoding variant surface antigens exhibit greater diversity and positive selection than other genes. These genes play an important role in *P. falciparum* malaria pathogenesis and in immune evasion by the malaria parasite. Studies in Africa have shown that severe malaria is associated with the ability of erythrocytes infected with the parasite to bind uninfected erythrocytes and form rosettes [39]. It is well-known that different members of these protein families bind to different adhesion receptors. The proteins are antigenically unique and switching of the individual PfEMP1 proteins during an infection is important for the maintenance of chronic infections. The enrichment of the positive Ka/Ks values on these genes is consistent with the previous reports that the high degree of diversity could help *P. falciparum* to fit the host immune system better. For example, STEVOR plays a role in creating antigenic diversity of schizont stage parasites, thereby adding additional complexity to the immunogenic properties of the infected red blood cell [40]. In our result, a total of 17 STEVOR genes have got valid value higher than 1 in Ka/Ks test, and 6 of them were under significant positive selection. These STEVOR genes offered enough modifications which enable the parasite to establish long-lasting chronic infection by evading antibody mediate immune recognition and splenic clearance. Similar selection pattern arose on *rif* genes family which contribute to the rosetting of *P. falciparum* mediated by blood antigen and help to express clonally variant antigens at the surface of the infected erythrocyte. Among all the 158 *rif* genes downloaded from plasmoDB, 66 genes have got valid value higher than 1, with 12 of them under significant positive selection. The signatures of positive selection suggested that the local environment complexity subjects CMB’s *P. falciparum* to more pressure for survival [26, 27].

## Conclusions

Our study assessed the genome sequences of six clinical isolates of *P. falciparum* in the CMB area. We found greater genetic diversity in CMB area and the positive selection signals in variant surface antigens genes. As gene flow is relatively unrestricted in East-south Asia, highly recombining populations of the *P. falciparum* are closely related, but markedly varying ecology and transmission intensity should cause distinct local selective pressures. Findings from this study provide more insight on the current epidemiological scenario of malaria in China. Lastly, our results will help deepen our understanding of *P. falciparum* evolution, and also provide the fundamental basis for further studies.

## Additional file

Additional file 1: Multilingual abstracts in the five official working languages of the United Nations. (PDF 899 kb)

## Abbreviations

CMB: China-Myanmar border
PCA: Principal component analysis
PfEMP1: *P. falciparum* erythrocyte membrane protein 1
RIFINs: Repetitive interspersed families of polypeptides
STEVOR: Subtelomeric variant open reading frame
VSAs: Variant surface antigens
