# Supplementary material for: Whole-genome sequencing and analysis of Plasmodium falciparum isolates from China-Myanmar border area
Source: Infect Dis Poverty. 2018 Nov 13;7:118. doi: 10.1186/s40249-018-0493-5 (PMC6240207; doi:10.1186/s40249-018-0493-5)
Supplement: Supplementary file 1 — Multilingual abstracts in the five official working languages of the United Nations. (PDF 899 kb) [file 40249_2018_493_MOESM1_ESM.pdf]

Translation of the abstract into the five official working languages of the United Nations

السلسلة الجينومية الكاملة وتحليل عزلات البلازمود المنجليمن المناطق الحدودية بين الصين وميانمار.

هاي مو شين، شين-بو تشن، يان-بينغ كوي، بن شو، كوكوفي كاسيني، انيولا مايكل ايب، يو وانغ، جون-هو تشن

مُلَخَّصٌ

الخلفية: أحرزت الصين تقدماً في مكافحة الملاريا وتهدف للقضاء على الملاريا في جميع أنحاء البلد، إلا أن تنفيذ التدخلات الفعالة على طول المناطق الحدودية تبقى مهمة جسيمة. يتم الإبلاغ عن الحالات الناجمة عن طفيل البلازمود المنجليالواردة من جنوب شرق آسيا بشكل متكرر خصوصاً في المنطقة الحدودية بين الصين وميانمار. ومع ذلك، فإن المعلومات حول التغير الجيني للبلازمود المنجليشحيحة في هذه المنطقة.

الطرق: تقرر هذه الدراسة أن البلازمود المنجلييعزل سلسلة الجينوم من ست عزلات إكلينيكية في المنطقة الحدودية بين الصين وميانمار. وعلاوة على ذلك، لقد قدرنا تنوع النوكليوتدات، ومقدِّر واترسون وقيمة تاجيما D لمعدل تحور الجينوم بأكلمه في نافذة الشريحة.

النتائج: تم محاذاة بياناتنا على 96.05 - 98.61% من الجينوم D73 المرجعي في أضعاف عالية من التغطية. وتُظهر نتائج تحليل المكون بأن البلازمود المنجليتتجمع بصورة عامة بحسب منشأهم الجغرافي. تم تحديد ما مجموعه 91 جين كاختيار إيجابي مع نسبة Ka/Ks أعلى بكثير من 1، ومعظمها كانت أسر متعددة الجينات تشفير المضادات سطحية متفاوتة (VSAs) مثل var, andrif stevor. إن تخصيب الإختيار الإيجابي على جينات VSA يدل أن تعقيد البيئة يعرض البلازمود المنجلي في المناطق الحدودية بين الصين وميانمار للمزيد من الضغط من أجل البقاء.

الاستنتاجات: ويدل بحثنا على أن التنوع الجيني الكبير في المنطقة الحدودية بين الصين وميانمار والاختيار الإيجابي يرسل إشارات في جينات VSA ، مما يسمح للبلازمود المنجلي بملائمة الجهاز المناعي المضيف جيداً ويزيد من صعوبة العلاج. وفي الوقت فسه، فإن النتائج المحرزة في هذه الدراسة ستوفر الأساس الجوهري للبحوث الجينومية السكانية للبلازمود المنجلي في المنطقة الحدودية بين الصين وميانمار.

Translated from English version into Arabic by Aya Kurdi, proofread by Mais Salsa, through

TRANSLATORS
WITHOUT BORDERS

中缅边境地区恶性疟原虫的全基因组测序及进化分析

沈海默, 陈绅波, 崔延冰, 徐斌, 卡森, 艾比, 王越, 陈军虎

摘要

引言：近年来中国在疟疾控制方面取得了巨大进展，但在边境地区实施有效干预仍然是一项艰巨的任务。从东南亚特别是中缅边境地区进入我国的输入性恶性疟病例时有报告。另外，这一地区的恶性疟原虫遗传变异信息尚不明确。

方法：本研究报道了中缅边境地区6个临床恶性疟病例的全基因组序列及其分析结果。我们使用滑框法，在全基因组层面上计算了Pi，Theta和Tajima’D值，并且统计了基因的Ka/Ks比值以确定其受选择的种类和程度。

结果：测序结果比对恶性疟原虫参考基因组3D7株达到了96.05−98.61%的覆盖度。主成分分析

表明，中缅边境恶性疟原虫呈现极强的地域特征。在恶性疟原虫全部5566个基因中，共3485个基因Ka/Ks值大于1，其中91个基因为显著正向选择，它们大多数为*var*、*rif*、*stevor*等基因，编码表面变异抗原（VSAs）的基因家族。在正向选择基因中，VSA基因的富集意味着环境和人类消除疟疾行为的复杂性使中缅边境地区的恶性疟承受了较大的生存压力。
结论：本研究表明中缅边境地区恶性疟原虫的遗传多样性程度较高，很多表面变异抗原基因处于正向选择压力之下，使得恶性疟原虫能够快速适应宿主的免疫系统，也加重了治疗的难度。同时，本研究的结果为边境地区恶性疟原虫的种群多样性和变异特征研究提供了基础依据。

Translated from English version into Chinese by Hai-Mo Shen

Séquençage du génome complet et analyse d'isolats de *Plasmodium falciparum* provenant de la région frontalière entre Chine et Myanmar

Hai-Mo Shen· Shen-Bo Chen· Yan-Bing Cui· Bin Xu· Kokouvi Kassegne· Eniola Michael Abe· Yue Wang· Jun-Hu Chen

Résumé
Contexte: La Chine a progressé dans la lutte contre le paludisme et vise l'élimination de la maladie dans tout le pays. Elle rencontre toutefois encore des difficultés énormes pour réaliser des interventions efficaces dans ses régions frontalières. Des cas de *Plasmodium falciparum* importés d'Asie du Sud-Est sont fréquemment rapportés, en particulier en provenance de la frontière entre la Chine et le Myanmar (Birmanie). Il n'y a cependant que peu d'informations disponibles sur la variabilité génétique de *P. falciparum* dans cette région.
Méthodes: La présente étude rapporte les séquences génomiques de six isolats cliniques de *P. falciparum* provenant de la frontière sino-birmane. Nous avons également estimé la diversité des nucléotides, la valeur de l'estimateur de Watterson et la valeur D de Tajima pour le taux global de mutations du génome sur une fenêtre glissante.
Résultats: Nos données concordent avec 96,05 à 98,61 % du génome de référence 3D7 avec des taux de couverture élevés. Le principal résultat de l'analyse des composants montre un regroupement de *P. falciparum* par origine géographique. Nous avons identifié au total 91 gènes qui forment une sélection positive où le rapport Ka/Ks était significativement supérieur à 1, la plupart étant des familles de plusieurs gènes codant des antigènes de surface variants (ASV) tels que *var*, *rif* et *stevor*. L'enrichissement de la sélection positive en gènes ASV suggère que la complexité de l'environnement exerce une pression de survie accrue sur les *P. falciparum* de la frontière sino-birmane.
Conclusions: Nos recherches suggèrent que la plus grande diversité génétique dans la région frontalière entre Chine et Myanmar et les signaux de sélection positive des gènes ASV permettent à *P. falciparum* de bien s'adapter au système immunitaire de ses hôtes et rend le traitement du paludisme encore plus difficile. Les résultats obtenus dans notre étude constitueront la base des recherches génomiques sur la population de *P. falciparum* dans la région de la frontière sino-birmane.

Translated from English version into French by Suzanne Assenat, proofread by Veronique Haour, through

TRANSLATORS
WITHOUT BORDERS

Полногеномное секвенирование и анализ изолятов *Plasmodium falciparum* в приграничных районах Китая с Мьянмой

Хай-Мо Шэнь, Шэнь-Бо Чэнь, Янь-Бин Цуй, Бинь Сюй, Кокуви Кассень, Эниола Михаэль Абе, Юэ Ван, Цзюнь-Ху Чэнь

Аннотация

История вопроса: Китай добился прогресса в борьбе с малярией и нацелен на ликвидацию малярии по всей стране, но осуществление эффективных вмешательств вдоль приграничных регионов остается огромной задачей. О случаях *Plasmodium falciparum* , завезённых из Юго-Восточной Азии, часто сообщалось, особенно в приграничном районе Китая с Мьянмой. Однако информация относительной генетической изменчивости *P. falciparum* по этому району недостаточна.

Методы: В данном отчёте об исследовании *P. falciparum* выделяется секвенирование генома шести клинических изолятов в исследуемом приграничном районе Китая с Мьянмой. Кроме того, мы измерили разнообразие нуклеотидов, оценку Уоттерсона и коэффициент D по тесту Тадзимы для полногеномной частоты мутации в окне микропрепарата.

Результаты: Наши данные были приведены в соответствие с 96,05 — 98,61% эталонного генома 3D7 в охватах с высокой кратностью наблюдений. Результат анализа основных компонентов показывает, что *P. falciparum* в основном группировались в соответствии с их географическим происхождением. В общей сложности 91 ген был идентифицирован как положительный отбор с отношением Ka / Ks значиельно выше 1, и большинство из них оказалось мультигенными семействами, кодирующими такие вариабельные поверхностные антигены (ВПА) как *var*, *rif* и *stevor*. Обогащение положительного отбора на генах ВПА подразумевало, что сложность среды подвергает *P. falciparum* приграничного района Китая с Мьянмой побуждает к усилению борьбы за выживание.

Выводы: Наши исследования показывают, что большее генетическое разнообразие в исследуемом приграничном районе и сигналы положительного отбора в генах ПВА позволяют паразиту *P. falciparum* хорошо приспособиться к иммунной системе хозяина и усугубить трудность лечения. Тем временем результаты, полученные в ходе этого исследования, будут служить основой для популяционных геномных исследований популяций паразита *P. falciparum* в приграничной зоне Китая с Мьянмой.

Translated from English version into Russian by Ira Kulinevych, proofread by Liudmila Tomanek, through

La secuenciación y análisis completo del genoma de las cepas de *Plasmodium falciparum* procedentes del área fronteriza China-Myanmar

Hai-Mo Shen· Shen-Bo Chen· Yan-Bing Cui· Bin Xu· Kokouvi Kassegne· Eniola Michael Abe· Yue Wang· Jun-Hu Chen

Resumen

Contexto: China progresó en el control del paludismo y tiene como objetivo erradicarlo completamente del país, pero implementar intervenciones efectivas en las regiones fronterizas sigue siendo una tarea difícil. Los casos de *Plasmodium falciparum* importados del sudeste de Asia son reportados frecuentemente, en especial en el área de la frontera entre China y Myanmar (CMB). Sin embargo, hay poca información sobre la variabilidad genética del P. falciparumen este área.

Métodos: Este informe del estudio de *P. falciparum* aisla la secuencia de genomas de seis aislados clínicos del área CMB. Además, calculamos la diversidad de nucleótidos, el estimador de Watterson y el valor D de Tajima para la tasa de mutación del genoma completo en el portaobjetos.

Resultados: Nuestros datos fueron ajustados al 96.05−98.61% del genoma 3D7 de referencia . Los resultados del análisis de los componentes principales muestran que el *P. falciparum* se agrupa generalmente según su zona geográfica. Un total of 91 genes fueron identificados como selección positiva con un cociente Ka/Ks notablemente mayor que 1, y la mayoría de ellos eran familias multigénicas que codifican antígenos de superficie variables (ASV) tales como *var*, *rif* y *stevor*. El enriquecimiento de la selección positiva en los genes ASV implica que la complejidad del ambiente somete al *P. falciparum* del área CMB a más presión para sobrevivir.

Conclusiones: Nuestro estudio sugiere que la mayor diversidad genética en el área CMB y las señales de selección positiva en los genes ASV , lo cual permite alP. falciparum adaptarse bien al sistema inmune del huésped ay empeorar la dificultad del tratamiento. Mientras tanto, los resultados obtenidos en este estudio brindarán una base fundamental para la investigación genómica del *P. falciparum* en la población del área CMB.

Translated from English version into Spanish by Luciana González, proofread by Noelia Bernárdez, through
